# Oxytocin attenuates hypothalamic injury-induced cognitive dysfunction by inhibiting hippocampal ERK signaling and Aβ deposition

**DOI:** 10.1038/s41398-024-02930-y

**Published:** 2024-05-25

**Authors:** Guangsen Wu, Yichao Ou, Zhanpeng Feng, Zhiwei Xiong, Kai Li, Mengjie Che, Songtao Qi, Mingfeng Zhou

**Affiliations:** Department of Neurosurgery, Institute of Brain Diseases, Nanfang Hospital of Southern Medical University, Guangzhou, China

**Keywords:** Epigenetics in the nervous system, Molecular neuroscience

## Abstract

In clinical settings, tumor compression, trauma, surgical injury, and other types of injury can cause hypothalamic damage, resulting in various types of hypothalamic dysfunction. Impaired release of oxytocin can lead to cognitive impairment and affect prognosis and long-term quality of life after hypothalamic injury. Hypothalamic injury-induced cognitive dysfunction was detected in male animals. Behavioral parameters were measured to assess the characteristics of cognitive dysfunction induced by hypothalamic–pituitary stalk lesions. Brains were collected for high-throughput RNA sequencing and immunostaining to identify pathophysiological changes in hippocampal regions highly associated with cognitive function after injury to corresponding hypothalamic areas. Through transcriptomic analysis, we confirmed the loss of oxytocin neurons after hypothalamic injury and the reversal of hypothalamic-induced cognitive dysfunction after oxytocin supplementation. Furthermore, overactivation of the ERK signaling pathway and β-amyloid deposition in the hippocampal region after hypothalamic injury were observed, and cognitive function was restored after inhibition of ERK signaling pathway overactivation. Our findings suggest that cognitive dysfunction after hypothalamic injury may be caused by ERK hyperphosphorylation in the hippocampal region resulting from a decrease in the number of oxytocin neurons, which in turn causes β-amyloid deposition.

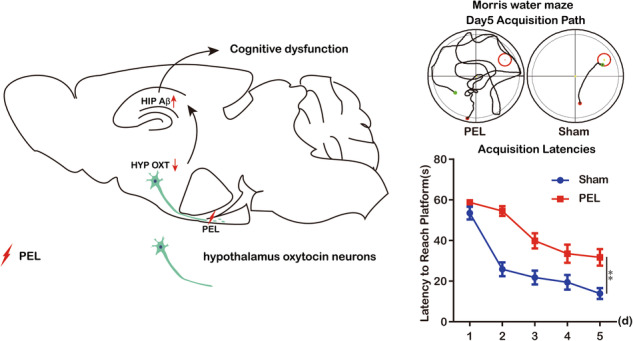

## Introduction

Oxytocin (OXT) has been found to have many functions outside of stimulating the mammary glands to produce milk, promoting uterine smooth muscle contractions during labor, and promoting motherhood. In addition to their endocrine roles, OXT and AVP are involved in a number of central processes, including learning and memory [1–4], anxiety [2, 3, 5], addiction [6, 7], hurt feelings [8], feeding behavior [9], and social recognition [10]. It has long been recognized that both OXT and AVP modulate learning, memory, and neuronal activity in the hippocampus. Early evidence from de Wied suggests that exogenous application of AVP provides long-term restoration of active avoidance behavior, which rapidly disappears in control pituitary ectomized rats [11]. This suggests an endogenous role for AVP in learning and memory. In rodents, OXT improved memory consolidation in a novel object recognition and passive avoidance paradigm [12–14]. Intraperitoneal injection of OXT contributes to cell proliferation, differentiation, and dendritic complexity of newborn neurons in the hippocampus. OXT can function more broadly than the modulation of social behaviors. OXT is involved in the integration of social and spatial information [15]. OXT enhances long-term synaptic plasticity and long-term memory via the MAP kinase cascade in the hippocampus [16]. OXT binds to hippocampal receptors to increase hippocampal LTP (long-term potentiation) in the hippocampus, thereby enhancing spatial memory in mice [17]. Many studies have shown that OXT plays an important role in cognitive processes such as the processing of sensory stimuli, social cognition, social memory and fear [18, 19] In human studies, autopsy results showed reduced expression of AVP immunoreactivity within the hippocampus, nucleus ambiguus, and pallidum in AD patients compared to controls [20]. Other autopsy studies of the human brain have provided evidence for a reduced number of AVP-expressing cells in the supraoptic nucleus of the optic chiasm in aging and AD patients [21]. OXT affects human processing of social information, including facial recognition memory. A single dose of intranasal OXT treatment improved the accuracy of face recognition without affecting the recognition of non-social stimuli [22]. The effect of OXT depends on the characteristics of the individual and the specificity of the situation to which he belongs. Thus, the effect of OXT on memory depends on their personality and feelings [23]. Intranasal OXT treatment negatively affects memory in individuals with high rates of attachment to others and positively affects memory in individuals with low rates of attachment [24]. The hypothalamus also secretes other neuronal hormones, including corticotropin-releasing hormone (CRH), gonadotropin-releasing hormone and prolactin, which are involved in modulation of anxiety, gonadal development, sexual activity, pregnancy, childbirth, and lactation, biological processes that are closely linked to oxytocin [25–27]. Mechanical injury, tumor compression, and surgical damage to the hypothalamus and related structures can cause impairment of other functions, including memory, social behavior, and emotion, which may be related to a deficiency in OXT caused by damage to the hypothalamic-pituitary system.

Various studies have revealed the specific manifestations of cognitive dysfunction, including in CNS disorders such as AD and PD, and identified the involvement of several processes such as β-amyloid (Aβ) deposition, inflammation, axonal degeneration and neuronal apoptosis [28–30]. Researchers have demonstrated in models of hypothalamic injury that the hypothalamic–pituitary system regulates water-sodium metabolism. However, no study has assessed the pattern of cognitive dysfunction after hypothalamic injury and validated the key pathways involved in hypothalamic injury-induced cognitive dysfunction.

In this study, we used a hypothalamic-pituitary stalk lesion model to comprehensively investigate the various behavioral changes after hypothalamic injury and determine the roles of several important molecules in different behaviors to identify the potential mechanisms of hypothalamic injury-induced cognitive dysfunction.

## Materials and methods

### Animals

All male C57BL/6 J mice were unrestricted access to feed and water in standard SPF experimental animal rooms at a controlled temperature and on a light-dark cycle. OXT-IRES-CRE mice were purchased from Shanghai Model Organisms. All experimental procedures were performed in accordance with the animal care guidelines of Southern Medical University.

### Hypothalamic injury model construction: Pituitary stalk electrical lesion (PEL) surgery

The mice were subjected to PEL surgery as described in a previous study [31–34]. Briefly, the 3D printed destruction knife is delivered to the pituitary stalk by stereotaxic positioning, and the pituitary stalk is destroyed by cathodic current.

### Immunofluorescence

Immunofluorescence assays were performed as previously described [34], Coronal brain sections were rinsed with PBS, blocked with 5% nonspecific antigen goat serum containing 0.5% Triton X-100 for 1 h at 37 °C, and incubated overnight at 4 °C with a specific primary antibody diluted in 5% goat serum containing 0.2% Triton X-100. The next day, after rinsed with PBS containing 0.2% Triton X-100 for 3 times, sections were incubated for 1 h at 37 °C with corresponding secondary antibodies conjugated with Alexa-488 or Alexa-594 (Thermo Fisher Scientific, Waltham, MA, USA) diluted in 5% goat serum containing 0.2% Triton X-100. After staining with 4-6-diamidino-2-phenylindole, all sections were slowly mounted on glass slides and cover glasses were slipped in mounting medium. Fluorescent images were captured with confocal microscope (LSM980; Zeiss, Oberkochen, Germany) using ZEN software (Blue edition, version 3.2). Labeled cells were counted using ImageJ software (NIH). For specific cell number counting, data were presented as the number of cells/slice. For double staining, data were presented as the ratio of double-positive cells to the single-positive cells.

### Three-chamber social interaction test

The three-chamber social interaction test consisted of three equally sized rooms (20 × 45 cm each) divided by clear Plexiglas, and with an access door between each compartment. The test occurred in three distinct stages: (1) acclimatization phase (baseline), where the test animal was placed in the central compartment and allowed to freely explore the entire empty maze for 10 min; (2) sociability phase, where an unfamiliar male (stranger 1) mouse was contained within a wire mesh container in an outer chamber of the maze and an identical clean and empty container was placed in the chamber at the opposite side of the arena. (3) social novelty phase, where another unfamiliar male (stranger 1) mouse was contained within another wire mesh container in an outer chamber of the maze and an identical clean and empty container was placed in the chamber at the opposite side of the arena. We also calculated the difference index, which is the difference between the time spent exploring the target (stranger 1 vs. null, stranger 2 vs. stranger 1) divided by the total time spent exploring both targets, as described previously [35].

### Morris water maze (MWM) test

All behavioral tests were performed during an active period of animals’ light cycle (07:00–19:00). Video analysis system (Shanghai Jiliang Software Technology Co., Ltd.) was used to observe and record the swimming pattern of each mouse. The pool was filled with water (21 ± 1 °C). An escape platform (6 cm in diameter) was placed in the pool and the top of the platform was 1.5 cm below the water surface. Mice were trained for 5 days with 4 trials/day (60 s/trial, 30 min inter-trial intervals). If the animal could not find the platform within 60 s, they were placed on the platform for 10 s. After 5 days of training, the hidden platform in the target quadrant was removed. On the sixth day, a probe test was administered. During the probe test, the platform was removed from the pool, and the mouse was allowed to swim freely for 30 s.

### The open field test

The Open Field Test (OFT) apparatus consists of four open areas (40 × 40 × 40 cm). Prior to testing, mice were placed in the laboratory and acclimatized to the chamber for 1 h. The mice were then placed individually in the center of the open areas and allowed to explore freely for 10 min. The time the mice spent in the center and the speed of their movements were recorded by a video tracking system (Shanghai Jiliang Software Technology Co., Ltd.). At the end of the test, the mice were returned to their cages. The OFT equipment was thoroughly cleaned with 75% ethanol at the end of each mouse test.

### Elevated plus maze test

The elevated plus maze (EPMT) is a platform consisting of a center stage (6 × 6 cm), two open arms (6 × 30 cm), and two closed arms (6 × 30 cm). The open arms and height were sufficient to induce anxiety in the mice. The mice were placed in the laboratory and acclimatized to the chamber for 1 h prior to EPMT. The mice were then placed individually on the center stage and allowed to explore freely for 5 min. The time the mice opened their arms and the number of times the mice entered the open arms were recorded by a video tracking system (Shanghai Jiliang Software Technology Co., Ltd.). At the end of the test, the mice were returned to their cages. The EPMT equipment was thoroughly cleaned with 75% ethanol at the end of each mouse test.

### Blood collection and ELISA assay

Mice were anesthetized with sodium pentobarbital (200 mg/kg intraperitoneally; Sigma-Aldrich,). Then, trunk blood was collected immediately by cardiac puncture into test tubes with anticoagulant and centrifuged at 1500 rpm for 15 min at 4 °C to isolate the plasma. Plasma fractions were stored at −80 °C for further analysis. To achieve accurate quantification of blood OXT contents, we performed additional sample extraction steps before Elisa testing as previously described [36, 37]. Briefly, solid phase extraction of the samples was performed using a 200 mg C18 Sep-Pak column (Thermo Fisher Scientific, CN). The column was equilibrated with 3 ml acetonitrile and then equilibrated twice with 3 ml 0.1% trifluoroacetic acid (TFA). Up to 0.6 ml of plasma was mixed with an equal volume of 0.1% TFA and centrifuged at 14,000 g for 20 min at 4 °C, and then the acidified and clarified plasma was applied to the column. The flow-through fraction was discarded and the column was washed once with 3 ml of 0.1% TFA and then twice with 3 ml of water. Oxytocin was eluted with 3 ml of 60% acetonitrile. The solvent was evaporated under a stream of nitrogen and the sample was completely dried by lyophilization. For immunoassays, the samples were reconstituted in 0.2 ml assay buffer provided with the Elisa kits. Elisa kits were purchased from LunChangShuoBiotech (LCSJZF20245). The extraction steps are as follows: 1. Equilibrate the kit and samples at room temperature (25–28 °C) for 120 min. 2. Set up the standard, sample and blank wells. (Single standard curve, 44 samples, samples double duplicate, 1 blank well). 3. Add 50 μL of different concentration of standard to each standard well. 4. 50 μL of the sample to be tested is added to the sample wells; 50 μL of sample diluent is added to the blank wells. 5. In all wells, add 100 μL of horseradish peroxidase (HRP)-labeled detection antibody to each well, seal the reaction wells with plate sealing film, and incubate at 37 °C in a water bath or thermostat for 60 min. 6. Discard the liquid, pat dry on absorbent paper, and wash the plate with a plate washer. 7. Add 50 μL each of substrate A and B to each well, and incubate at 37 °C away from light for 15 min. 8. Add 50 μL of termination solution to each well, and measure the OD value of each well at 450 nm within 15 min.

### Stereotactic surgery

The mice were deeply anesthetized with 75 mg/kg sodium pentobarbital and were mounted on a stereotaxic frame (RWD Life Sciences Co., Shenzhen, China). An approximately 1 × 1 mm^2^ piece of skull was removed at the following coordinates: −0.55 AP, +/−0.30 ML, and −5.10 DV (PVN); −0.55 AP, +/−1.25 ML, and −6.10 DV (SON), −1.9 AP, +/−1 ML, and − 2 DV (hippocampus) −1.9 AP, 0 ML, and – 6.1 DV (pituitary stalk). Virus (200 nl) was infused at 50 nl/min using a glass micropipette connected to a Hamilton syringe, backfilling the tubing with mineral oil. After infusion, the micropipette was elevated 0.05 mm, kept in place for 5 min to allow for virus diffusion and then slowly removed.

### Drug

OXT (HY-17571A; MedChemExpress) was diluted in saline and administered intraperitoneally (1 mg/kg/d for i.p [38, 39]. 50 ng for hippocampal stereotactic injection [40, 41]). AVP (ZY604698A; Shanghai Zeye Biotechnology Co. LTD) was diluted in saline and administered intraperitoneally (0.1 mg/kg/d [39, 42]). Atosiban(ATO; HY-17572A; MedChemExpress;) was diluted in saline and administered intraperitoneally (1.5 mg/kg/d for i.p. [43], 50 ng for hippocampal stereotactic injection [41]) Clozapine N-oxide (CNO) (K948133-100 mg; ShangHai D&B Biological Science and Technology Co. Ltd) was diluted in 5% DMSO and administered intraperitoneally (3 mg/kg/d).

### Western blotting

Western blotting assays were performed as previously described [33].

### Antibodies

The following primary antibodies were used: rabbit anti-AVP (AB1565; Millipore), rabbit anti-OXT (ab212193; Abcam, rabbit anti-ERK (51068-1-AP; Proteintech), rabbit anti-p-ERK (28733-1-AP; Proteintech), rabbit anti-OXTR (23045-1-AP; Proteintech), rabbit anti-Aβ (A17911; Abclonal), rabbit anti-AMPK (A1229; Abclonal), rabbit anti -p-AMPK (AP1002; Abclonal), rabbit anti-JNK (A0288; Abclonal), rabbit anti-p-JNK (AP0631; Abclonal),and rabbit anti-GAPDH (AC001; Abclonal).

### Virus

The following viruses were used: AAV2/9-mOXT: Promoter-hM3Dq-mCherry-WPRE-pA (packaged by Taitool Bioscience) and AAV2/9-CAG-DIO-taCaspase3-TEVp-WPRE-pA (S0236-9 Taitool Bioscience).

### RNA preparation for high-throughput RNA sequencing

To obtain an adequate amount of RNA for high-throughput RNA sequencing, hypothalamic tissue containing the SON and PVN was collected from mice after PEL surgery as described in our previous study [34].

### Gene expression analysis

RNA-seq reads were aligned to the reference genome using Hisat2 v2.0.5. Feature Counts v1.5.0-p3 was used to calculate the number of reads mapped to each gene. Differential expression analysis was performed using the DESeq2 method (version 1.10.1). The resulting *p* values were adjusted using the Benjamini and Hochberg method to control for the false discovery rate (<0.05). Genes with *p* < 0.05 (determined by DESeq2) between different groups were considered differentially expressed.

### Functional enrichment analysis of differentially expressed genes (DEGs)

DEGs between the groups (PEL vs. Sham) with adjusted *p* < 0.05 were further analyzed using the publicly available bioinformatics software Database for Annotation, Visualization and Integrated Discovery. Clustering analysis based on the Benjamini method revealed that DEGs with an *p* < 0.05 were enriched in several Gene Ontology (GO) terms. The gene set enrichment analysis (GSEA) and GO enrichment analysis results were further visualized by the R package clusterProfiler [44].

### Statistical analysis

No formal sample size estimation or randomization was performed, and all available KO mice and WT littermates were included in the experiments. Investigators were not blinded to allocation and outcome analysis. The Kolmogorov‒Smirnov and Shapiro‒Wilk normality tests were used to assess the distribution of the data. Unpaired two-tailed Student’s *t*-test was used to analyze normally distributed data. Mann–Whitney test was performed in case of not normally distributed data. One or two-way analysis of variance (ANOVA) with Tukey’s or Bonferroni multiple comparison post-hoc test was performed where applicable. All statistical analyses were performed using SPSS software (version 23.0, IBM, Armonk, NY, USA). The error bars in all plots indicate the SEMs. *p* < 0.05 was considered significant. All reported *p*-values are two-tailed. Statistical significance was presented using the following rules: *p < 0.05, **p < 0.01, ***p < 0.001, ns- not significant

## Results

### Characteristics of water-electrolyte metabolism dysfunction after hypothalamic injury

To carry out an animal study of hypothalamic injury, we constructed a PEL model of hypothalamic injury using a three-dimensional printed knife in combination with electrolytic injury via a transparietal approach. As shown in. S1A, we performed PEL surgery on day 0. In addition, we performed RNA-seq analysis of hypothalamic cell nuclei from PEL mice to identify potentially relevant changes in gene expression patterns. The mice were housed individually in metabolic cages after modeling, and metrics related to water-electrolyte metabolism, including DWC, DUV, and USG, were measured to assess the degree of water-electrolyte metabolism dysfunction in the organisms.

**Fig. 1:**
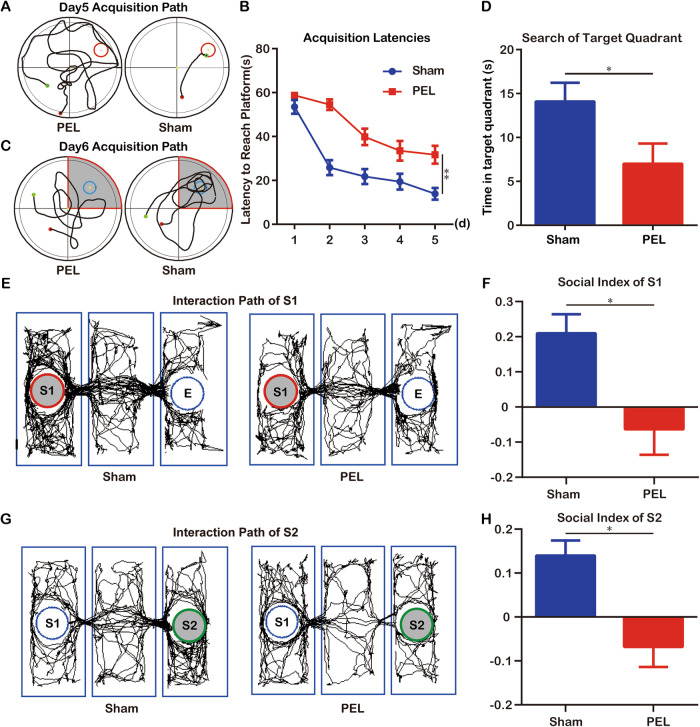
PEL-induced cognitive dysfunction. **A** Trajectories of PEL mice (*n* = 6) and Sham mice (*n* = 6) in the MWM place navigation experiment. **B** Quantitative analysis of latency in the MWM place navigation experiment. (F (1, 46) = 50.95, *p* < 0.01.). **C** Trajectories of PEL (*n* = 6) mice and Sham (*n* = 6) mice in the MWM spatial exploration experiment. The shaded part is the area around the platform, and the activity time of mice in the shaded part is calculated. **D** Quantitative analysis of swimming time around the platform in the MWM spatial exploration experiment. (*t* = 2.245, df = 10, *p* < 0.05.). **E** Trajectories of PEL (*n* = 6) and Sham (*n* = 6) mice in the three-chambers social experiment (S1), The shaded area is the cage where S1 is located. **F** Quantification of the social index of S1. (*t* = 3.012, df = 10, *p* < 0.05). **G** Trajectory of PEL (*n* = 6) and Sham (*n* = 6) mice in the three-chambers social experiment (S2), The shaded area is the cage where S2 is located. **H** Quantification results of S2 social index. (*t* = 3.666, df = 10, *p* < 0.05.). Data are analyzed by 2-way ANOVA (**B**) and unpaired *t* test (**D**, **F**, **H**). data are expressed as mean ± SEM. Compared with Sham, *P < 0.05, **P < 0.01. MWM Morris Water Maze, S1 Stranger1, S2 Stranger2.

Uremia occurred in three phases in the hypothalamic injury model mice: the acute phase, which occurred on the first postoperative day and was characterized by high DWC (Fig. S1B) and DUV (Fig. S1C) and low USG (Fig. 1D); the second phase, which occurred on days 2–4 postoperatively and was characterized by a significant decrease followed by a gradual increase in DWC and DUV and a significant increase followed by a gradual decrease in USG; and the chronic phase, which occurred on days 5–28 postoperatively and was characterized by persistently high DWC, high DUV and low USG. Plasma AVP levels also decreased significantly (Fig. S1E). In addition, immunofluorescence staining of sagittal sections demonstrated damage to the pituitary stalk and a diminished pituitary AVP signal after PEL surgery (Fig. S1F).

### Cognitive dysfunction after hypothalamic injury

In the open field test (OFT), there was no significant difference in speed between the Sham group mice and the PEL mice, suggesting that the PEL treatment does not affect the locomotor ability of mice. (Fig. S2A, B) To explore whether cognitive function is impaired in mice after hypothalamic injury, we conducted a water maze experiment (MWM test) to evaluate spatial memory in mice. In the first five days, during the place navigation experiment, the mean latency to find the hidden platform was significantly lower for mice in the Sham-operated group than for those in the hypothalamic injury group (Fig. 1B). On the sixth day, during the spatial exploration experiment, mice in the Sham-operated control group spent a significantly longer mean time in the area around the platform than mice in the hypothalamic injury group (Fig. 1D). In the three-chamber socialization test, which was used to evaluate the social interaction ability of the mice, the social index for stranger mouse 1 significantly lower for mice in the hypothalamic injury group than for mice in the Sham-operated control group, suggesting social dysfunction in mice in the hypothalamic injury group (Fig. 1F). Similarly, the social index for stranger mouse 2 was significantly higher in the Sham-operated control group than in the hypothalamic injury group (Fig. 1H), suggesting that the social memory of mice in the hypothalamic injury group was decreased. However, in the OFT, there was no significant difference in the time in the central zone between the two groups of mice (Fig. S2A, C) proving that the PEL treatment did not affect the anxiety and stress levels of the mice, and this result was further corroborated by the elevated plus maze test (EPM), which showed that there was no significant difference in the open-arm entries and the time in open-arms between the two groups (Fig. S2D–F). In summary, hypothalamic injury results in cognitive dysfunction characterized by spatial memory impairment, decreased social skills, and impaired social memory. These results suggest that hypothalamic injury-induced cognitive dysfunction is characterized by impaired spatial memory and social skills.

### AVP and OXT neuron loss after hypothalamic injury

**Fig. 2:**
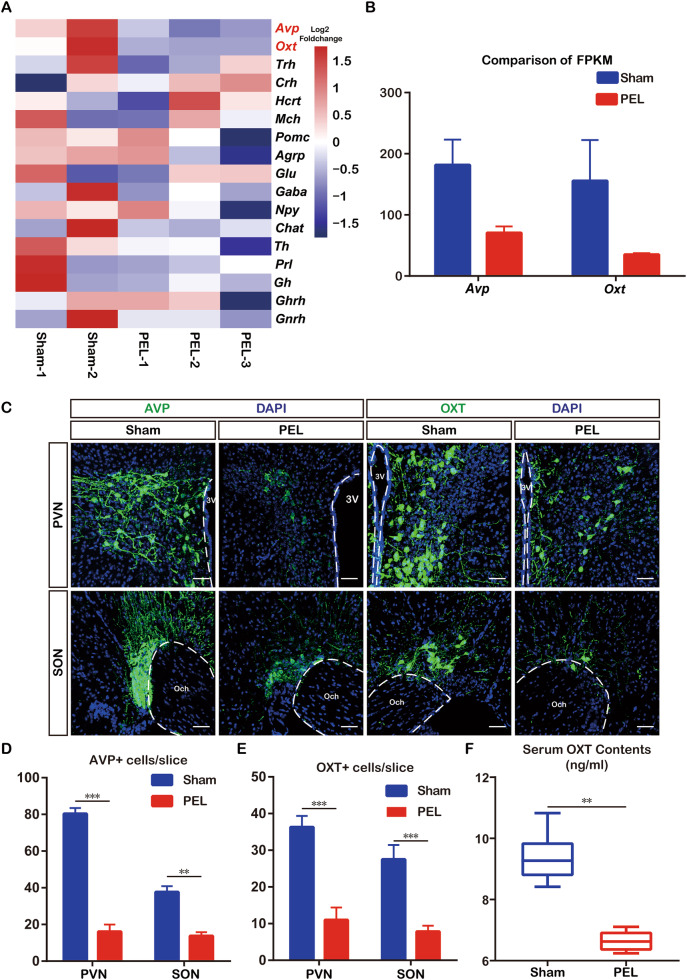
RNA-seq analysis showing hypothalamic AVP, OXT neuron loss. Heat map of the hypothalamic neurons) DEGs was significantly lower between PEL and Sham groups (*n* = 3 for PEL, *n* = 2 for Sham, adjusted *p*-value [p-adj] <0.05) (**A**). Quantitative results of decreased AVP and OXT FPKM values after PEL surgery. hypothalamic SON and PVN AVP, OXT neuron loss in mice after PEL surgery (**B**). OXT neuronal loss was verified (**C**). **D** Quantification results of residual AVP neurons (*n* = 6, PVN: *t* = 32.06, df = 10, *p* < 0.001; SON: *t* = 15.58, df = 10 *p* < 0.01). **E** Quantification results of residual OXT neurons in (*n* = 6, PVN: *t* = 13.65, df = 10 *p* < 0.001; SON: *t* = 11.33, df = 10, *p* < 0.001). Plasma OXT levels were measured by ELISA (Sham *n* = 6, PEL *n* = 6, *t* = 7.548, df = 10, *p* < 0.01) (**F**). Scale bar, 20 μm. Data were analyzed by *t*-test (**B**, **D**–**F**). Data are expressed as mean ± SEM. Compared to Sham, **P < 0.01, ***P < 0.001. AVP arginine vasopressin, OXT oxytocin, DEG differentially expressed gene, SON supraoptic nucleus, PVN paraventricular nucleus, DAPI diamino-2-phenylindole, 3 V third ventricle, Och optic chiasma.

To explore the underlying mechanisms of hypothalamic injury-induced cognitive dysfunction, we extracted RNA from the mouse hypothalamus, including the SON and PVN, for high-throughput sequencing. Raw RNA sequence data are available from the public GEO database. Through RNA-seq analysis, we identified DEGs between the PEL and Sham groups (499 up-regulated and 7 down-regulated). Among the markers of hypothalamic neurons, AVP and OXT were the two most markedly downregulated genes in the PEL_10d group compared to the Sham group, indicating abnormal *Avp* and *Oxt* expression and synthesis in the hypothalamic–pituitary system (Fig. 2A). Therefore, we further performed AVP and OXT staining in PEL mice to verify the pathophysiological changes in the SON and PVN (There is a outlier sample in Sham group, so it was not analyzed Fig. S2I).

As shown in Fig. 2C, the number of AVP neurons per SON slice was reduced from 37.7 in the Sham group to 13.8 in the PEL group (Fig. 2D). The number of AVP neurons per PVN slice was also significantly reduced from 80.3 in the Sham group to 16.2 in PEL group (Fig. 2D). The number of OXT neurons per PVN slice was reduced from 36.3 in the control group to 11 in the PEL group (Fig. 2E). The number of OXT neurons per SON slice was also significantly reduced from 27.5 in the control group to 7.8 in the PEL group (Fig. 2E). We further explored whether the CRH axis was affected, and as Fig. S2G shown, there was no significant reduction in the number of CRH neurons in the PEL group relative to Sham (Fig. S2H). ELISA suggested that plasma OXT levels decreased from 9.370 pg/ml in the control group to 6.642 pg/ml in the PEL group (Fig. 2F). We next examined the content of OXT in the hippocampus, and the expression of the PEL group was significantly decreased relative to that of the Sham group (Fig. S2J, K), suggesting that PEL induced a systemic decrease in OXT expression.

### Exogenous supplementation with OXT relieves hypothalamic injury-induced cognitive dysfunction

**Fig. 3:**
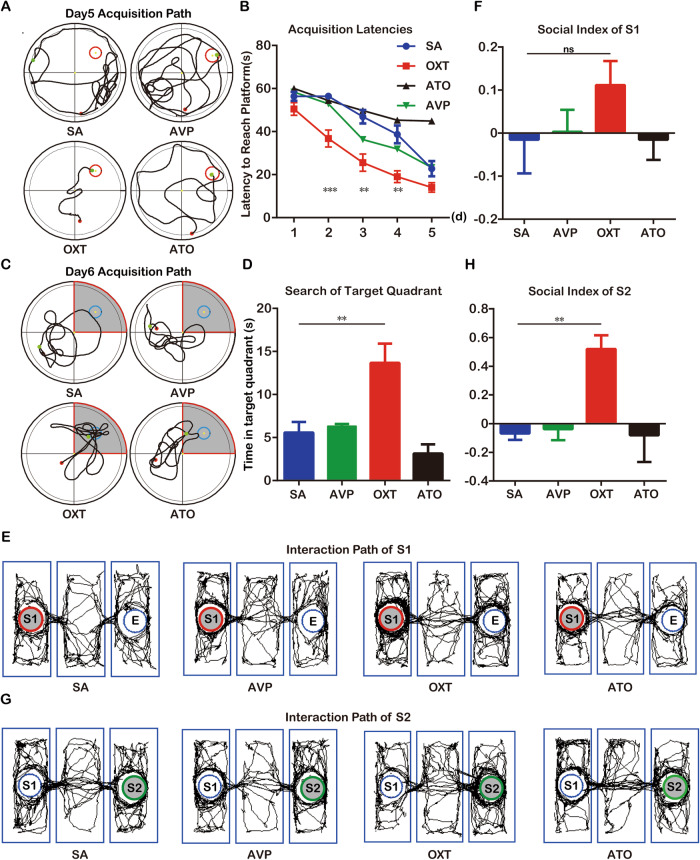
Exogenous supplementation of OXT attenuated hypothalamic-derived cognitive dysfunction. **A** Trajectories of PEL-treated group (*n* = 6), AVP-supplemented group (*n* = 6), OXT-supplemented group (*n* = 6), and ATO group (*n* = 6) in the MWM place navigation experiment. **B** Quantitative analysis of latency in the MWM place navigation experiment. (F (3, 92) = 23.80 *p* < 0.01). **C** Trajectories of PEL-treated group (*n* = 6), AVP-supplemented group (*n* = 6), OXT-supplemented group (*n* = 6), and ATO group (*n* = 6) in the MWM spatial exploration experiment. The shaded part is the area around the platform, and the activity time of mice in the shaded part is calculated. **D** Quantitative analysis of swimming time around the platform in the MWM spatial exploration experiment. (F (3, 20) = 10.56, *p* < 0.01). **E** Trajectories diagrams of PEL-treated group (*n* = 6), AVP-supplemented group (*n* = 6), OXT-supplemented group (*n* = 6), and ATO group (*n* = 6) in the three-chambers social experiment (S1). The shaded area is the cage where S1 is located. **F** Quantitative results of S1 social index. (F (3, 20) = 1.054, *p* = 0.3908). **G** Trajectories of PEL-treated group (*n* = 6), AVP-supplemented group (*n* = 6), OXT-supplemented group (*n* = 6), and ATO group (*n* = 6) in the three-chambers social experiment (S2). The shaded area is the cage where S2 is located. **H** Quantification results of S2 social index. F (3.000, 10.13) = 6.611, *p* < 0.01. Data were analyzed by two-way ANOVA (**B**), 1-way ANOVA followed by LSD multiple comparisons test (**D**, **F**) and Brown-Forsythe ANOVA test (**H**). Data are expressed as mean ± SEM. Compared to PEL-treated group, **P < 0.01. MWM Morris Water Maze, S1 Stranger1, S2 Stranger2, OXT oxytocin, ATO Atosiban.

Based on the sequencing results, we explored the role played by AVP and OXT in hypothalamic injury-induced cognitive dysfunction. We administered exogenous OXT or AVP at days 15–21 after PEL surgery, and cognitive function tests were then performed. In the place navigation experiment of the MWM test, the mean latency of mice in the saline control group was significantly higher than that of mice in the OXT-treated group but not significantly different from that of mice in the AVP-treated group. Furthermore, the therapeutic effect of OXT could be inhibited by the specific OXT receptor antagonist atosiban (ATO) (Fig. 3B). In the spatial exploration experiment, the mice in the saline control group spent significantly less time in the area around the platform than the mice in the OXT-treated group, but there was no significant difference between the saline control group and the AVP-treated group. The therapeutic effect of OXT could also be inhibited by ATO (Fig. 3D). Exogenous OXT supplementation after hypothalamic injury contributed to the recovery of spatial memory in mice. In the three-chamber socialization test, the social index for stranger 1 was higher in the OXT-treated mice than in the remaining three groups, but the difference was not statistically significant (Fig. 3F). The social index for stranger 2 was significantly higher in the OXT-treated group than in the saline control group; the therapeutic effect of OXT was inhibited by ATO, and AVP had no therapeutic effect (Fig. 3H). Further, we supplemented OXT by stereotactic injection into the hippocampus after PEL, followed by MWM experiments, and the results were shown: In the place navigation experiment of the MWM test, the mean latency of mice in the PEL group was significantly higher than that of mice in the OXT-treated group (Fig. S3C). In the spatial exploration experiment, the mice in the PEL group spent significantly less time in the area around the platform than the mice in the OXT-treated group (Fig. S3E). These results suggested that OXT treatment partially alleviates hypothalamic injury-induced cognitive dysfunction, and the hippocampus is the target brain region of the therapeutic effects of OXT.

### Activation of residual OXT neurons after PEL surgery can ameliorate hypothalamic injury-derived cognitive dysfunction

**Fig. 4:**
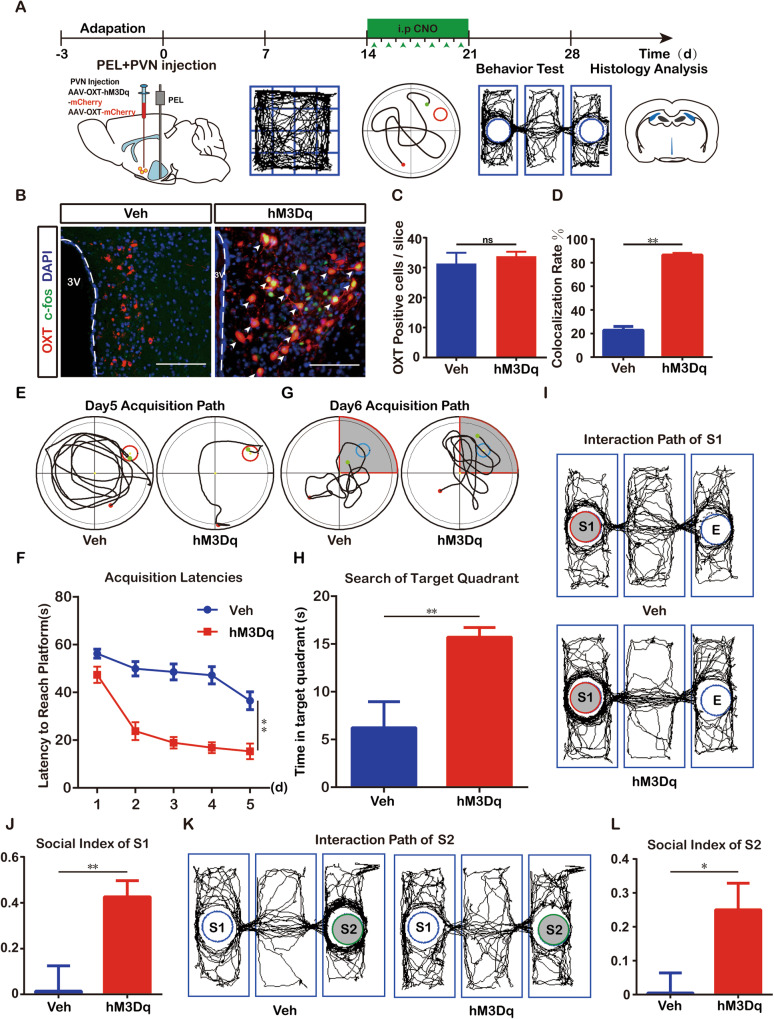
Activation of endogenous OXT neurons after PEL also attenuates hypothalamic-derived cognitive dysfunction. **A** Experimental study protocol for activation of endogenous OXT neurons after PEL. **B** Validation of activation of endogenous OXT neurons in mice with PVN OXT neurons activated after PEL surgery. **C** Quantitative analysis of the number of OXT neurons in the Veh (*n* = 6) and CNO (*n* = 6) groups. (*t* = 1.569, df = 10, *p* = 0.1477). **D** Quantitative analysis of the co-staining rate of OXT neurons with c-fos in the Veh (*n* = 6) and CNO (*n* = 6) groups. (Mann–Whitney *U* = 0, *p* < 0.01). **E** Trajectories of Veh (*n* = 6) mice and CNO (*n* = 6) mice in the MWM place navigation experiment. **F** Quantitative analysis of latency in the MWM place navigation experiment. (F (1, 46) = 91.72, *p* < 0.01). **G** Trajectories of Veh (*n* = 6) mice and CNO (*n* = 6) mice in the MWM spatial exploration experiment. The shaded part is the area around the platform, and the activity time of mice in the shaded part is calculated. **H** Quantification of activity time around the platform in the MWM spatial exploration experiment. (*t* = 3.248, df = 10, *p* < 0.01). **I** Trajectories of Veh (*n* = 6) and CNO (*n* = 6) mice in the three-chambers social experiment (S1). The shaded area is the cage where S1 is located. **J** Quantification of the social index of S1. (Mann–Whitney *U* = 0, *p* < 0.01). **K** Trajectory of Veh (*n* = 6) mice and CNO (*n* = 6) mice in the three-chambers social experiment (S2), The shaded area is the cage where S2 is located. **L** Quantitative results of S2 social index. (t = 2.493, df = 10, *p* < 0.05). Data were analyzed by t test (**C**); Mann–Whitney test (**D**, **J**); two-way ANOVA (**F**) and t-test (**H**, **L**). Data are expressed as mean ± SEM. Compared to Veh, *P < 0.05, **P < 0.01. Scale bar, 20 μm. MWM: Morris Water Maze; S1: Stranger1; S2: Stranger2; CNO: Clozapine N-oxide; 3 V, third ventricle；Veh: vehicle.

Furthermore, we activated endogenous OXT neurons by stereotaxic injection of AAV2/9-mOXT: Promoter-hM3Dq-mCherry-WPRE-pA or AAV2/9-mOXT: Promoter-mCherry-WPRE-pA into the PVN combined with CNO treatment after PEL surgery, as shown in Fig. 4A. There was no difference in the number of OXT neurons between the vehicle and CNO groups (Fig. 4C), but the colocalization rate of OXT with c-fos in the CNO group was significantly higher (Fig. 4D). Then, cognitive function tests were performed. The mean latency of mice in the CNO group was significantly lower than that of mice in the vehicle group in the place navigation experiment of the MWM test (Fig. 4F). Mice in the CNO group spent significantly more time in the area around the platform than mice in the vehicle group in the spatial exploration experiment (Fig. 4H). These findings demonstrated that activation of endogenous OXT neurons after hypothalamic injury helped to prevent the impairment of spatial memory in mice. In the three-chamber socialization test, the social index of mice in the CNO group was significantly higher than that of mice in the vehicle group (S1: Fig. 4J; S4: Fig. 4L), suggesting that social function and social memory were recovered in mice in the hypothalamic injury group after activation of endogenous OXT neurons.

### OXT plays an important role in cognitive function

**Fig. 5:**
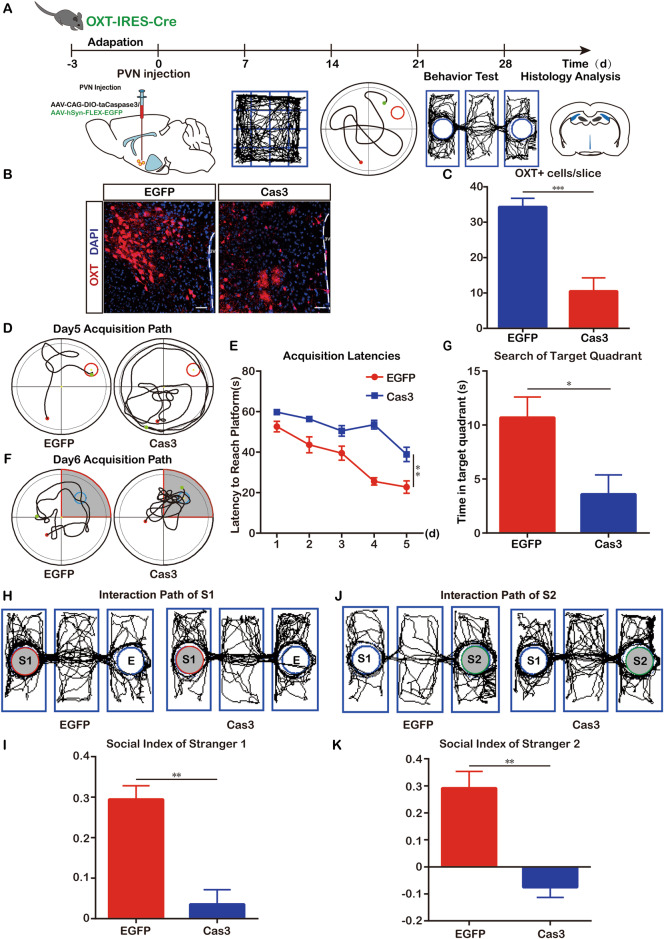
Ablation of OXT neurons leads to hypothalamic-derived cognitive dysfunction. **A** Experimental study protocol for ablation of OXT neurons. **B** OXT neuronal loss was verified after Cas3 virus injection. **C** Quantification results of residual OXT neurons in (n = 6, t = 13.00, df=10, *p* < 0.001.). **D** Trajectory of EGFP (n = 6) mice and Cas3 (n = 6) mice in the MWM place navigation experiment. **E** Quantitative analysis of latency in the MWM place navigation experiment. (F (1, 46) = 41.21, *p* < 0.01). **F** Trajectories of EGFP (n = 6) mice and Cas3 (n = 6) mice in the MWM spatial exploration experiment. The shaded part is the area around the platform, and the activity time of mice in the shaded part is calculated. **G** Quantitative analysis of activity time around the platform in the MWM spatial exploration experiment. (t = 6.663, df=10, *p* < 0.05). **H** Trajectories of EGFP (n = 6) and Cas3 (n = 6) mice in the three-chambers social experiment (S1). The shaded area is the cage where S1 is located. **I** Quantification of social index in S1. (t = 5.253, df=10, *p* < 0.01). **J** Trajectory of EGFP (n = 6) mice and Cas3 (n = 6) mice in the three-box experiment (S2). The shaded area is the cage where S2 is located. **K** Quantitative results of S2 social index. (t = 5.085, df=10, *p* < 0.01). Data were analyzed by two-way ANOVA (**E**) and *t*-test (**C**, **G**, **J**, **K**). Data are expressed as mean ± SEM. Compared to EGFP, *P < 0.05, **P < 0.01. Scale bar, 20 μm. MWM: Morris Water Maze; S1: Stranger1; S2: Stranger2; Cas3: caspase3.

To further explore the role of OXT in cognitive function, we induced apoptosis of OXT neurons in OXT-ires-Cre mice by injecting AAV-CAG-DIO-taCaspase3 and assessed cognitive function, as shown in Fig. 5A. In the place navigation experiment of the MWM test, the mean latency of mice in the Cas3 group was significantly higher than that of mice in the EGFP group (Fig. 5C); in the spatial exploration experiment, mice in the Cas3 group spent significantly less time in the area around the platform than mice in the control group (Fig. 5E). In the three-chamber socialization test, the socialization index for stranger 1 and stranger 2 were significantly lower in the Cas3 group (0.036 and −0.076, respectively) than in the EGFP group (0.295 and 0.291, respectively) (S1: Fig. 5J; S4: Fig. 5K), suggesting that OXT neurons play an active role in cognitive function.

### Promotion of ERK signaling pathway activity based on RNA-Seq analysis

**Fig. 6:**
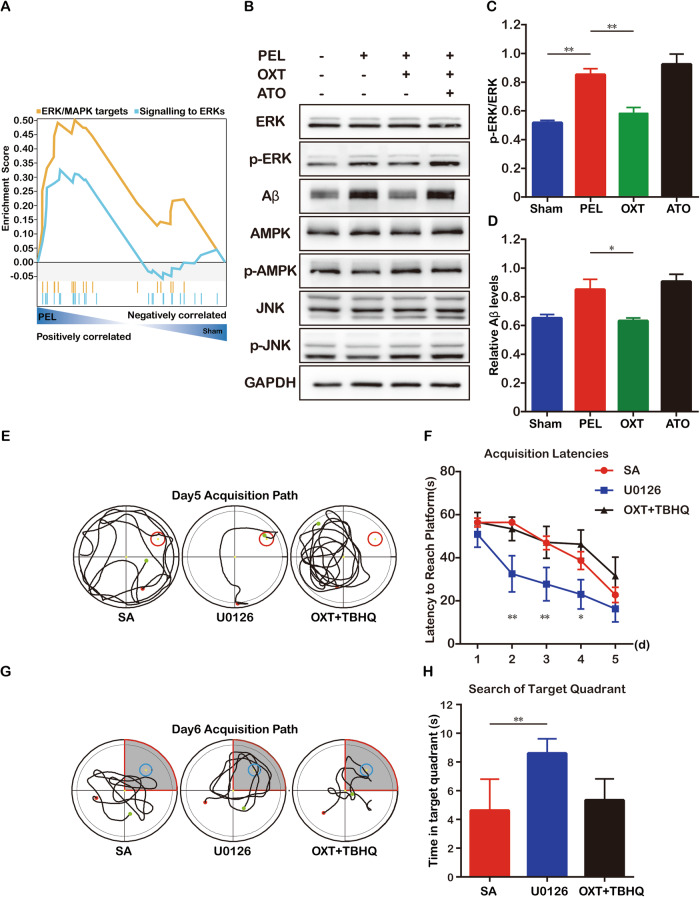
Enrichment analysis identifies hippocampal ERK signaling pathway involved in hypothalamic-derived cognitive dysfunction. **A** results of GSEA analysis show upregulation of ERK signaling pathway in the PEL group (n = 6) compared to the Sham group (n = 6). Immunoblotting confirmed the elevated expression of p-ERK in hippocampal tissues of mice after PEL surgery (**B**, **C** F (3.000, 12.18) = 109.2, *p* < 0.01). Immunoblotting confirmed elevated Aβ expression in hippocampal tissues of mice after PEL surgery, and OXT reduced Aβ expression (**B**, **D** Kruskal–Wallis statistic=17.95, *p* < 0.01). Trajectory diagram of PEL group (n = 6), U0126 supplemented group (n = 6) and TBHQ group (n = 6) mice in the MWM place navigation experiment (**E**). Quantitative analysis of latency in the MWM place navigation experiment (**F**) (F (2, 69) = 22.05, *p* < 0.01). Trajectory diagrams of PEL group (n = 6), supplemented U0126 group (n = 6), and TBHQ group mice (n = 6) in the MWM spatial exploration experiment. The shaded part is the area around the platform, and the activity time of mice in the shaded part is calculated. **G** Quantitative analysis of activity time around the platform in the MWM spatial exploration experiment (**H**). (F (2, 15) = 16.57 *p* < 0.01). Data were analyzed by 1-way ANOVA followed by LSD multiple comparisons test followed by LSD multiple comparisons test (**C**, **H**), Kruskal–Wallis test (**D**) and two-way ANOVA (**F**). Data are expressed as mean ± SEM. Compared to Sham, **P < 0.01. PEL, pituitary stalk electrical damage; GO gene ontology; GSEA gene set enrichment analysis, MWM Morris Water Maze.

The hippocampus is thought to be strongly associated with cognition and memory. Therefore, we used hippocampal tissue for RNA-seq analysis and found that OXTR expression was elevated in the PEL group compared with the control group, but the difference was not statistically significant (Fig. S4A). Furthermore, we found that the expression of components of the ERK signaling pathway (Fig. 6A), which is considered the canonical pathway via which OXT signals through OXTR, was significantly increased in the PEL group. Western blotting revealed that OXTR expression was significantly elevated in the hippocampus of mice in the injury group (Fig. S4C), ERK phosphorylation was significantly increased (Fig. 6C), and Aβ expression was significantly increased (Fig. 6D), consistent with previous studies showing that hyperphosphorylation of ERK in the hippocampus leads to Aβ deposition, which in turn causes cognitive dysfunction. These findings suggest that the number of OXT neurons and OXT secretion are reduced after hypothalamic injury, leading to increased hippocampal ERK phosphorylation, which could lead to elevated Aβ expression and thus cognitive dysfunction.

### Hyperphosphorylation of hippocampal ERK mediates hypothalamic injury-induced cognitive function

Furthermore, we assessed cognitive function after intraperitoneal injection of the ERK signaling pathway inhibitor U0126 and activator TBHQ following PEL surgery. To verify the effects of U0126 and TBHQ, we performed Western blotting, which showed a significant decrease in ERK phosphorylation and decrease in Aβ expression after U0126 treatment and a significant increase in ERK phosphorylation and elevated Aβ expression after TBHQ treatment (ERK: Fig. S4E; Aβ: Fig. S4F). The MWM test also showed that similar to OXT treatment, intraperitoneal injection of U0126 alone after PEL surgery improved the spatial memory of PEL mice, while TBHQ blocked the therapeutic effect of OXT (localization cruise: Fig. 6F; spatial exploration: Fig. 6H;).

## Discussion

### Dysfunction after hypothalamic injury

The hypothalamus plays a vital role in the endocrine system [45]. In neurosurgery, especially tumor resection surgery and other surgeries in regions near the hypothalamus or involving the pituitary stalk, the secretion of trans-pituitary hormones such as AVP and OXT is easily impaired; the pathological changes that occur after impaired AVP secretion were fully explored in our previous studies [33, 34]. Other neurobehavioral and psychiatric abnormalities include deficits in cognition, memory, emotional control and social functioning, mood disorders, and apathy, among others [46, 47]. The most common cognitive deficits associated with hypothalamic injury are paracrine-related situational memory deficits. They range in severity from a mild inability to learn, maintain, and later recall new information beyond the short term to now-rare cases of severe Korsakoff-like deficits characterized by severe amnesia, dissociation, and disorientation to time and place. Situational memory deficits after hypothalamic injury may occur because the hypothalamus is an important component of the Papez memory loop [48], which includes the hippocampus. These deficits in cognitive function may be related to a decrease in the levels of OXT, another neuropeptide, caused by damage to the hypothalamic–pituitary system.

In clinical practice, 17–27% of patients with preoperative craniopharyngioma and almost all postoperative patients exhibit symptoms similar to those of our PEL model when the pituitary stalk is sacrificed during tumor resection [49, 50]. In this study, we constructed a model of hypothalamic injury induced by complete damage to the pituitary stalk, which is also applicable to the study of hypothalamic neuronal loss due to PEL injury, as the pituitary stalk receives axonal output from large cell neurons in the SON and PVN. PEL mice exhibit cognitive dysfunction characterized by reduced spatial memory and social skills, which is consistent with the typical clinical signs of patients after hypothalamic injury. We studied alterations in cognitive function, morphology and molecular biology induced by hypothalamic–pituitary stalk injury in mice, initially confirmed the correlation between hypothalamic–pituitary stalk injury and water-electrolyte metabolism dysfunction and cognitive dysfunction, and preliminarily explored the characteristics of hypothalamic injury-induced water-electrolyte metabolism dysfunction and cognitive dysfunction. The changes resulting from hypothalamic injury caused by tumor compression or neurosurgical injury were partially recapitulated, indicating that this mouse model of hypothalamic–pituitary stalk injury is a reliable animal model for the study of hypothalamic injury.

### Treatment of hypothalamic injury-induced cognitive dysfunction by OXT

OXT was originally thought to be a regulator of childbirth and lactation. However, recent studies have elucidated the effects of OXT on social behaviors such as partnering and maternal care as well as aggression, anxiety, fear, and interpersonal trust. Many subsequent studies have also demonstrated its involvement in the regulation of social behavior in all vertebrates, and it has been proposed for the treatment of a range of neuropsychiatric disorders characterized by deficits in the social domain [51–53]. OXT synthesis and secretion require an intact hypothalamic–pituitary system, and hypothalamic injury involving the hypothalamic–pituitary system can cause impaired OXT synthesis and release, resulting in OXT deficiency and related neuroendocrinological disorders. The main manifestations are cognitive dysfunction, including memory dysfunction and decreased social skills. In this study, we performed transcriptomic analysis of PEL model mice and subsequently investigated specific changes in gene expression patterns and cognitive deficits in PEL model mice to elucidate the underlying regulatory mechanisms. Considering that PEL surgery mainly damages AVP and OXT neuronal axons passing through the pituitary stalk, we focused mainly on AVP and OXT neurons and found that the cell bodies of AVP and OXT MCN and AVP and OXT neuronal fibers were eliminated from the hypothalamic–pituitary system and that antidiuretic peptides and OXT were absent from the systemic circulation. This is also consistent with our results of transcriptomic analysis of PEL model rats. These changes could be responsible for the cognitive dysfunction observed in mice after hypothalamic injury. The central OXT system is a potential therapeutic target for alleviating cognitive dysfunction in many psychiatric disorders.

There are several ways to promote OXT system activity: the most common approach is direct OXT supplementation, which was utilized in initial studies on the therapeutic effect of OXT in ASD [54].In patients with ASD, intravenous high-dose OXT was found to both improve the retention of social information and reduce stereotyped repetitive behaviors; direct OXT supplementation was found to markedly improve social and perceptual abilities, increasing the perception of socially important information such as emotion, empathy, facial information, and responses to biological actions [55]. It also promotes interpersonal relationships through socially reinforced learning, trust and attachment enhancement [56]. The second approach is to activate endogenous OXT neurons to produce more neuronal peptides by optogenetic or chemogenetic methods; these methods have been widely used in cognition-related studies and have been shown to increase OXT levels in the neuropil.

We directly administered OXT or AVP to replenish neuropeptides lost after hypothalamic injury and demonstrated that supplementation with OXT but not AVP after hypothalamic injury partially alleviated cognitive dysfunction, leading to substantial improvements in learning, spatial memory, social skills, and social memory. Furthermore, we activated residual OXT neurons after hypothalamic injury by a chemogenetic approach, and the results showed that hypothalamic injury-induced cognitive dysfunction was also partially improved after OXT neurons were activated. Next, we specifically knocked out OXT neurons in the hypothalamus with a virus that induced apoptosis and showed that the cognitive dysfunction observed after OXT neuron ablation was similar to that observed after PEL surgery, further validating the importance of OXT for cognitive function, improving the understanding of the pathophysiological processes associated with hypothalamic injury, and providing new targets for further prevention and treatment of cognitive dysfunction.

OXT in the brain signals through OXTR, a 7-transmembrane G protein-coupled receptor. In the hippocampus, OXTR appears to be coupled to G-coupled Q/11 proteins that can activate multiple signaling cascades with various downstream effects [57, 58]. The major OXTR signaling cascade involves the G-type Q/11 pathway, which can regulate the mitogen-activated protein kinase (MAPK) cascade and ultimately the activity of extracellular signal-regulated kinase 1/2 (ERK1/2) [59].

### Relevance of the ERK signaling pathway and Aβ deposition in hypothalamic injury-induced cognitive dysfunction

The ERK/MAPK pathway has been extensively studied and found to play a key role in neurodevelopment as well as in learning, memory, synaptic plasticity, and spinal dynamics [60]. The functional importance of ERK/MAPK signaling is also reflected in the fact that mutations in ERK1/2 that lead to hyperactivation cause a range of syndromic and nonsyndromic neurodevelopmental disorders, including impaired cognitive function characterized by memory impairment with social deficits [61, 62]. It has been demonstrated that hyperphosphorylation of ERK in the hippocampus leads to Aβ deposition [63], and β-amyloid (Aβ) forms oligomeric aggregates that interact with membrane receptors, causing a neuropathic cascade that ultimately leads to synaptic dysfunction and neuronal death [64]. Amyloid plaques and neurofibrillary tangles are mainly found in the hippocampus and other limbic brain regions involved in learning and memory and emotion regulation [65–67]. In particular, the hippocampus is particularly susceptible to amyloid toxicity resulting in dementia-like changes [68, 69].

Previous studies have demonstrated that multiple signaling pathways, including the ERK1/2, P38, AMPK, c-jun, and jnk signaling pathways, are involved in OXT-mediated regulation of learning and cognitive functions, including spatial memory, social skills, and emotion [70]. Our RNA-seq experiment demonstrated that the expression of genes enriched in the ERK signaling pathway was increased in hippocampal regions associated with cognition and memory after hypothalamic injury. Furthermore, Western blotting verified that hippocampal ERK1/2 phosphorylation levels changed with OXT levels after hypothalamic injury and OXT treatment, and Aβ levels changed accordingly. Taken together, our results showed that ERK1/2 hyperphosphorylation and thus Aβ deposition due to reduced OXT levels are associated with cognitive dysfunction after hypothalamic injury. However, more studies are needed to investigate the specific mechanism of Aβ production in the hippocampus after hypothalamic injury.

## Conclusion

Our findings suggest that cognitive dysfunction after hypothalamic injury may be caused by ERK hyperphosphorylation in the hippocampal region resulting from a decrease in the number of OXT neurons, which in turn causes Aβ deposition. The present study provides a new mechanism of the role for OXT in cognition and uncovers ERK signaling in memory modulation.

### Supplementary information

Supplementary Material

## Data Availability

Original RNA seq data can be acquired from the pubic GEO database (GSE229838). Other data that support the findings of this study are available from the corresponding author upon reasonable request.

## References

[1] Alescio-Lautier B, Soumireu-Mourat B (1998). Role of vasopressin in learning and memory in the hippocampus. Prog Brain Res.

[2] Caldwell HK, Lee HJ, Macbeth AH, Young WS (2008). Vasopressin: behavioral roles of an “original” neuropeptide. Prog Neurobiol.

[3] Lee HJ, Macbeth AH, Pagani JH, Young WS (2009). Oxytocin: the great facilitator of life. Prog Neurobiol.

[4] de Wied D, Diamant M, Fodor M (1993). Central nervous system effects of the neurohypophyseal hormones and related peptides. Front Neuroendocrinol.

[5] Huang T, Guan F, Licinio J, Wong ML, Yang Y (2021). Activation of septal OXTr neurons induces anxiety- but not depressive-like behaviors. Mol Psychiatry.

[6] Bowen MT, Neumann ID (2017). Rebalancing the addicted brain: oxytocin interference with the neural substrates of addiction. Trends Neurosci.

[7] Sarnyai Z, Kovács GL (2014). Oxytocin in learning and addiction: from early discoveries to the present. Pharm Biochem Behav.

[8] Tracy LM, Georgiou-Karistianis N, Gibson SJ, Giummarra MJ (2015). Oxytocin and the modulation of pain experience: Implications for chronic pain management. Neurosci Biobehav Rev.

[9] Sabatier N, Leng G, Menzies J (2013). Oxytocin, feeding, and satiety. Front Endocrinol (Lausanne).

[10] Ferguson JN, Aldag JM, Insel TR, Young LJ (2001). Oxytocin in the medial amygdala is essential for social recognition in the mouse. J Neurosci.

[11] Drago F, Bohus B (1986). Hyperprolactinaemia alleviates behavioral alterations of rats with hereditary hypothalamic diabetes insipidus (Brattleboro strain). Physiol Behav.

[12] Gard PR, Naylor C, Ali S, Partington C (2012). Blockade of pro-cognitive effects of angiotensin IV and physostigmine in mice by oxytocin antagonism. Eur J Pharm.

[13] Kovács GL, Bohus B, Versteeg DH, de Kloet ER, de Wied D (1979). Effect of oxytocin and vasopressin on memory consolidation: sites of action and catecholaminergic correlates after local microinjection into limbic-midbrain structures. Brain Res.

[14] Gaffori OJ, De Wied D (1988). Bimodal effect of oxytocin on avoidance behavior may be caused by the presence of two peptide sequences with opposite action in the same molecule. Eur J Pharm.

[15] Sánchez-Vidaña DI, Chan NM, Chan AH, Hui KK, Lee S, Chan HY (2016). Repeated treatment with oxytocin promotes hippocampal cell proliferation, dendritic maturation and affects socio-emotional behavior. Neuroscience.

[16] Rice MA, Hobbs LE, Wallace KJ, Ophir AG (2017). Cryptic sexual dimorphism in spatial memory and hippocampal oxytocin receptors in prairie voles (Microtus ochrogaster). Horm Behav.

[17] Insel TR, Young L, Wang Z (1997). Central oxytocin and reproductive behaviours. Rev Reprod.

[18] Ferguson JN, Young LJ, Hearn EF, Matzuk MM, Insel TR, Winslow JT (2000). Social amnesia in mice lacking the oxytocin gene. Nat Genet.

[19] Stoop, R, C Hegoburu, E van den Burg. New opportunities in vasopressin and oxytocin research: a perspective from the Amygdala. in Annual Review of Neuroscience, Vol 38, SE Hyman, Editor; 2015. p. 369–88. 10.1146/annurev-neuro-071714-033904 26154981

[20] Raskind MA, Peskind ER, Lampe TH, Risse SC, Taborsky GJ, Dorsa D (1986). Cerebrospinal fluid vasopressin, oxytocin, somatostatin, and beta-endorphin in Alzheimer’s disease. Arch Gen Psychiatry.

[21] Mazurek MF, Beal MF, Bird ED, Martin JB (1986). Vasopressin in Alzheimer’s disease: a study of postmortem brain concentrations. Ann Neurol.

[22] Rimmele U, Hediger K, Heinrichs M, Klaver P (2009). Oxytocin makes a face in memory familiar. J Neurosci.

[23] Bartz JA, Zaki J, Bolger N, Ochsner KN (2011). Social effects of oxytocin in humans: context and person matter. Trends Cogn Sci.

[24] Frankiensztajn LM, Gur-Pollack R, Wagner S (2018). A combinatorial modulation of synaptic plasticity in the rat medial amygdala by oxytocin, urocortin3 and estrogen. Psychoneuroendocrinology.

[25] Hemberg M (2017). Summing up the parts of the hypothalamus. Nat Neurosci.

[26] Haller J, Makara GB, Barna I, Kovacs K, Nagy J, Vecsernyes M (1996). Compression of the pituitary stalk elicits chronic increases in CSF vasopressin, oxytocin as well as in social investigation and aggressiveness. J Neuroendocrinol.

[27] Guo L, Qi YJ, Tan H, Dai D, Balesar R, Sluiter A (2022). Different oxytocin and corticotropin-releasing hormone system changes in bipolar disorder and major depressive disorder patients. EBioMedicine.

[28] Bachmann D, Roman ZJ, Buchmann A, Zuber I, Studer S, Saake A (2022). Lifestyle affects amyloid burden and cognition differently in men and women. Ann Neurol.

[29] Berron D, van Westen D, Ossenkoppele R, Strandberg O, Hansson O (2020). Medial temporal lobe connectivity and its associations with cognition in early Alzheimer’s disease. Brain.

[30] Edate S, Albanese A (2015). Management of electrolyte and fluid disorders after brain surgery for pituitary/suprasellar tumours. Horm Res Paediatr.

[31] Feng Z, Ou Y, Zhou M, Wu G, Ma L, Bao Y (2018). A rat model for pituitary stalk electric lesion-induced central diabetes insipidus: application of 3D printing and further outcome assessments. Exp Anim.

[32] Feng Z, Ou Y, Zhou M, Wu G, Ma L, Zhang Y (2018). Functional ectopic neural lobe increases GAP-43 expression via PI3K/AKT pathways to alleviate central diabetes insipidus after pituitary stalk lesion in rats. Neurosci Lett.

[33] Zhou M, Ou Y, Wu G, Li K, Peng J, Wang X (2022). Transcriptomic analysis reveals that activating transcription factor 3/c-Jun/Lgals3 axis is associated with central diabetes insipidus after hypothalamic injury. Neuroendocrinology.

[34] Zhou M-F, Feng Z-P, Ou Y-C, Peng J-J, Li K, Gong H-D (2019). Endoplasmic reticulum stress induces apoptosis of arginine vasopressin neurons in central diabetes insipidus via PI3K/Akt pathway. Cns Neurosci Therapeutics.

[35] Chen S, He L, Huang AJY, Boehringer R, Robert V, Wintzer ME (2020). A hypothalamic novelty signal modulates hippocampal memory. Nature.

[36] Szeto A, McCabe PM, Nation DA, Tabak BA, Rossetti MA, McCullough ME (2011). Evaluation of enzyme immunoassay and radioimmunoassay methods for the measurement of plasma oxytocin. Psychosom Med.

[37] Tabak BA, Leng G, Szeto A, Parker KJ, Verbalis JG, Ziegler TE (2023). Advances in human oxytocin measurement: challenges and proposed solutions. Mol Psychiatry.

[38] Choe KY, Bethlehem RAI, Safrin M, Dong H, Salman E, Li Y (2022). Oxytocin normalizes altered circuit connectivity for social rescue of the Cntnap2 knockout mouse. Neuron.

[39] Hicks C, Ramos L, Reekie T, Misagh GH, Narlawar R, Kassiou M (2014). Body temperature and cardiac changes induced by peripherally administered oxytocin, vasopressin and the non-peptide oxytocin receptor agonist WAY 267,464: a biotelemetry study in rats. Br J Pharm.

[40] Oliveira VEM, Lukas M, Wolf HN, Durante E, Lorenz A, Mayer AL (2021). Oxytocin and vasopressin within the ventral and dorsal lateral septum modulate aggression in female rats. Nat Commun.

[41] Zhu W, Ding Z, Zhang Z, Wu X, Liu X, Zhang Y (2021). Enhancement of oxytocin in the medial prefrontal cortex reverses behavioral deficits induced by repeated ketamine administration in mice. Front Neurosci.

[42] Tan O, Musullulu H, Raymond JS, Wilson B, Langguth M, Bowen MT (2019). Oxytocin and vasopressin inhibit hyper-aggressive behaviour in socially isolated mice. Neuropharmacology.

[43] Hofmann J, Huber C, Novak B, Schreckenbach M, Schubert CF, Touma C (2021). Oxytocin receptor is a potential biomarker of the hyporesponsive HPA axis subtype of PTSD and might be modulated by HPA axis reactivity traits in humans and mice. Psychoneuroendocrinology.

[44] Yu G, Wang L-G, Han Y, He Q-Y (2012). clusterProfiler: an R package for comparing biological themes among gene clusters. OMICS.

[45] Burbridge S, Stewart I, Placzek M (2016). Development of the neuroendocrine hypothalamus. Compr Physiol.

[46] Bauer HG (1954). Endocrine and other clinical manifestations of hypothalamic disease; a survey of 60 cases, with autopsies. J Clin Endocrinol Metab.

[47] Maria Pascual J, Prieto R, Castro-Dufourny I, Mongardi L, Rosdolsky M, Strauss S (2018). Craniopharyngiomas primarily involving the hypothalamus: a model of neurosurgical lesions to elucidate the neurobiological basis of psychiatric disorders. World Neurosurg.

[48] Vann SD (2010). Re-evaluating the role of the mammillary bodies in memory. Neuropsychologia.

[49] Mueller HL (2020). The diagnosis and treatment of craniopharyngioma. Neuroendocrinology.

[50] Mueller HL, Merchant TE, Warmuth-Metz M, Martinez-Barbera J-P, Puget S. Craniopharyngioma. Nat Rev Dis Primers. 2019;5. 10.1038/s41572-019-0125-9 31699993

[51] Alharfi IM, Stewart TC, Foster J, Morrison GC, Fraser DD (2013). Central diabetes insipidus in pediatric severe traumatic brain injury. Pediatr Crit Care Med.

[52] Dulac C, O’Connell LA, Wu Z (2014). Neural control of maternal and paternal behaviors. Science.

[53] Rilling JK, Young LJ (2014). The biology of mammalian parenting and its effect on offspring social development. Science.

[54] Hollander E, Novotny S, Hanratty M, Yaffe R, DeCaria CM, Aronowitz BR (2003). Oxytocin infusion reduces repetitive behaviors in adults with autistic and Asperger’s disorders. Neuropsychopharmacology.

[55] Domes G, Lischke A, Berger C, Grossmann A, Hauenstein K, Heinrichs M (2010). Effects of intranasal oxytocin on emotional face processing in women. Psychoneuroendocrinology.

[56] Hurlemann R, Patin A, Onur OA, Cohen MX, Baumgartner T, Metzler S (2010). Oxytocin enhances amygdala-dependent, socially reinforced learning and emotional empathy in humans. J Neurosci.

[57] Rae M, Lemos Duarte M, Gomes I, Camarini R, Devi LA (2022). Oxytocin and vasopressin: signalling, behavioural modulation and potential therapeutic effects. Br J Pharmacol.

[58] Froemke RC, Young LJ. Oxytocin, neural plasticity, and social behavior. in Annual Review of Neuroscience, Vol 44, 2021, Roska B, Zoghbi HY and editors. 2021. p. 359–81. 10.1146/annurev-neuro-102320-102847 PMC8604207 33823654

[59] Ye C, Cheng M, Ma L, Zhang T, Sun Z, Yu C (2022). Oxytocin nanogels inhibit innate inflammatory response for early intervention in Alzheimer’s disease. Acs Appl Mater Interfaces.

[60] Thomas GM, Huganir RL (2004). Mapk cascade signalling and synaptic plasticity. Nat Rev Neurosci.

[61] Tidyman WE, Rauen KA (2016). Pathogenetics of the RASopathies. Hum Mol Genet.

[62] Borrie SC, Brems H, Legius E, Bagni C. Cognitive dysfunctions in intellectual disabilities: The Contributions of the Ras-MAPK and PI3K-AKT-mTOR Pathways. in Annual Review of Genomics and Human Genetics, Vol 18, A Chakravarti and ED Green, Editors; 2017. p. 115-42. 10.1146/annurev-genom-091416-035332 28859574

[63] Kim E, Kim H, Jedrychowski MP, Bakiasi G, Park J, Kruskop J (2023). Irisin reduces amyloid-β by inducing the release of neprilysin from astrocytes following downregulation of ERK-STAT3 signaling. Neuron.

[64] Nistico R, Pignatelli M, Piccinin S, Mercuri NB, Collingridge G (2012). Targeting synaptic dysfunction in Alzheimer’s disease therapy. Mol Neurobiol.

[65] Holtzman DM, Morris JC, Goate AM. Alzheimer’s disease: the challenge of the second century. Sci Transl Med. 2011;3. 10.1126/scitranslmed.3002369 PMC3130546 21471435

[66] Gomez-Isla T, Price JL, McKeel DW, Morris JC, Growdon JH, Hyman BT (1996). Profound loss of layer II entorhinal cortex neurons occurs in very mild Alzheimer’s disease. J Neurosci.

[67] Murray C, Viehman A, Lippa CF (2006). The corpus callosum in Pick’s disease, Alzheimer’s disease, and amyotrophic lateral sclerosis: gliosis implies possible clinical consequence. Am J Alzheimer’s Dis Other Dement.

[68] Mu Y, Gage FH. Adult hippocampal neurogenesis and its role in Alzheimer’s disease. Mol Neurodegener. 2011;6. 10.1186/1750-1326-6-85 PMC3261815 22192775

[69] Hollands C, Bartolotti N, Lazarov O. Alzheimer’s disease and hippocampal adult neurogenesis; exploring shared mechanisms. Front Neurosci. 2016;10. 10.3389/fnins.2016.00178 PMC4853383 27199641

[70] Jurek B, Neumann ID (2018). The oxytocin receptor: from intracellular signaling to behavior. Physiol Rev.

