# Supplementary material for: Oxytocin attenuates hypothalamic injury-induced cognitive dysfunction by inhibiting hippocampal ERK signaling and Aβ deposition
Source: Transl Psychiatry. 2024 May 25;14:208. doi: 10.1038/s41398-024-02930-y (PMC11127955; doi:10.1038/s41398-024-02930-y)
Supplement: Supplementary file 1 — Supplementary Material [file 41398_2024_2930_MOESM1_ESM.docx]

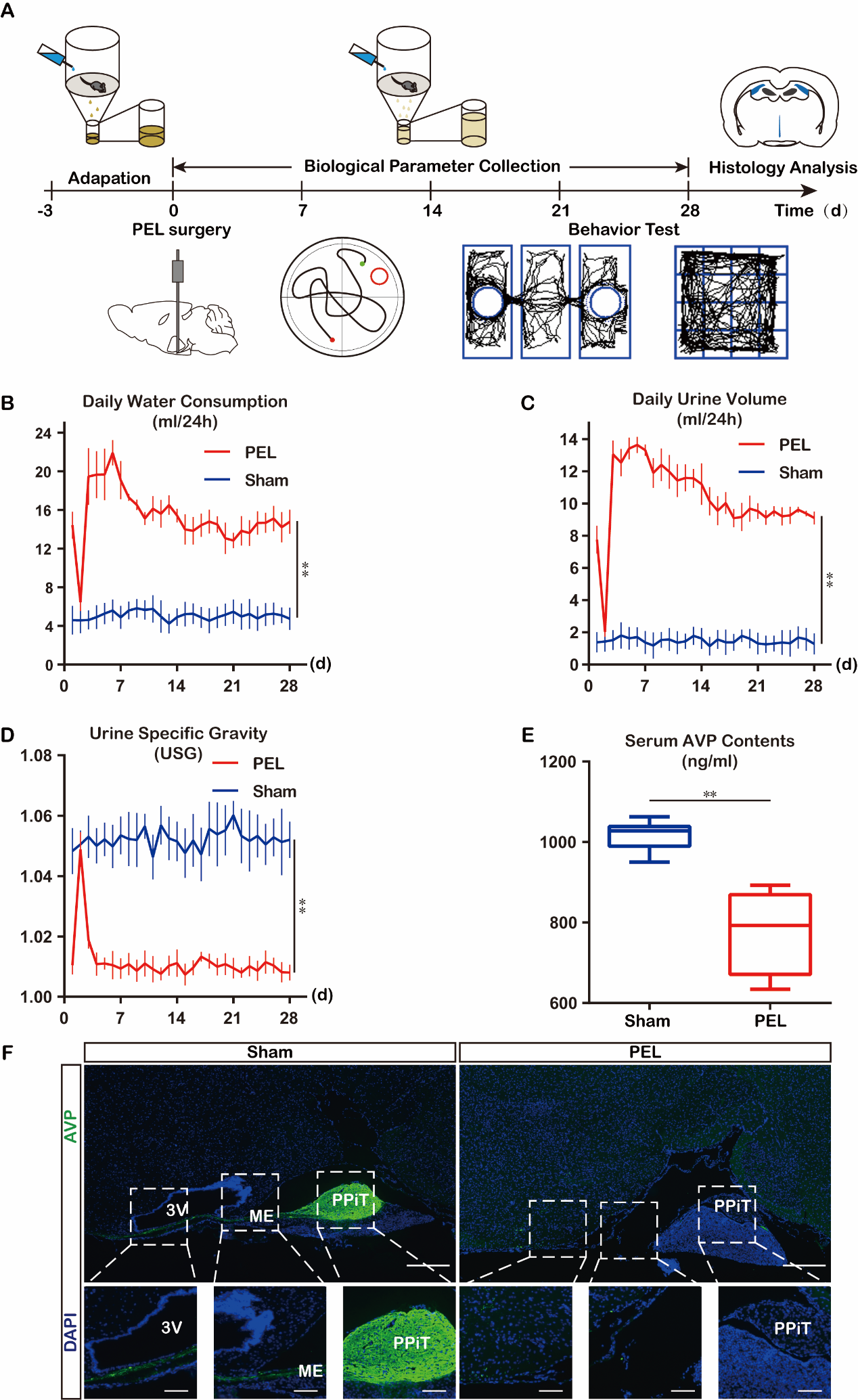


Figure S1. typical CDI phenotype induced by hypothalamic PEL.

(A) The entire experimental protocol of this study. DWC (B F (1, 10) = 5152,*P*＜0.01), DUV (C H= 242.5855382, *P*＜0.01) and USG (D H= 238.1449454, *P*＜0.01) in PEL mice and Sham mice (n=6). Serum AVP levels were measured by ELISA (n=6) (E Mann-Whitney U= 0 *P*＜0.01 ) (F) Disruption of pituitary stalk continuity and loss of AVP neurons in PEL mice. Scale bars, 50 μm for low magnification images and 20 μm for high magnification images. Data are analyzed by unpaired t test (B–E). Data are expressed as mean ± SEM. Compared with Sham, ***P* <0.01. PEL: pituitary stalk electrical injury; DWC: daily water consumption; DUV: daily urine volume; USG: urine specific gravity; CDI, central diabetes insipidus; AVP, arginine vasopressin; OXT: oxytocin; 3V, third ventricle; ME, median eminence; PPiT, posterior pituitary.


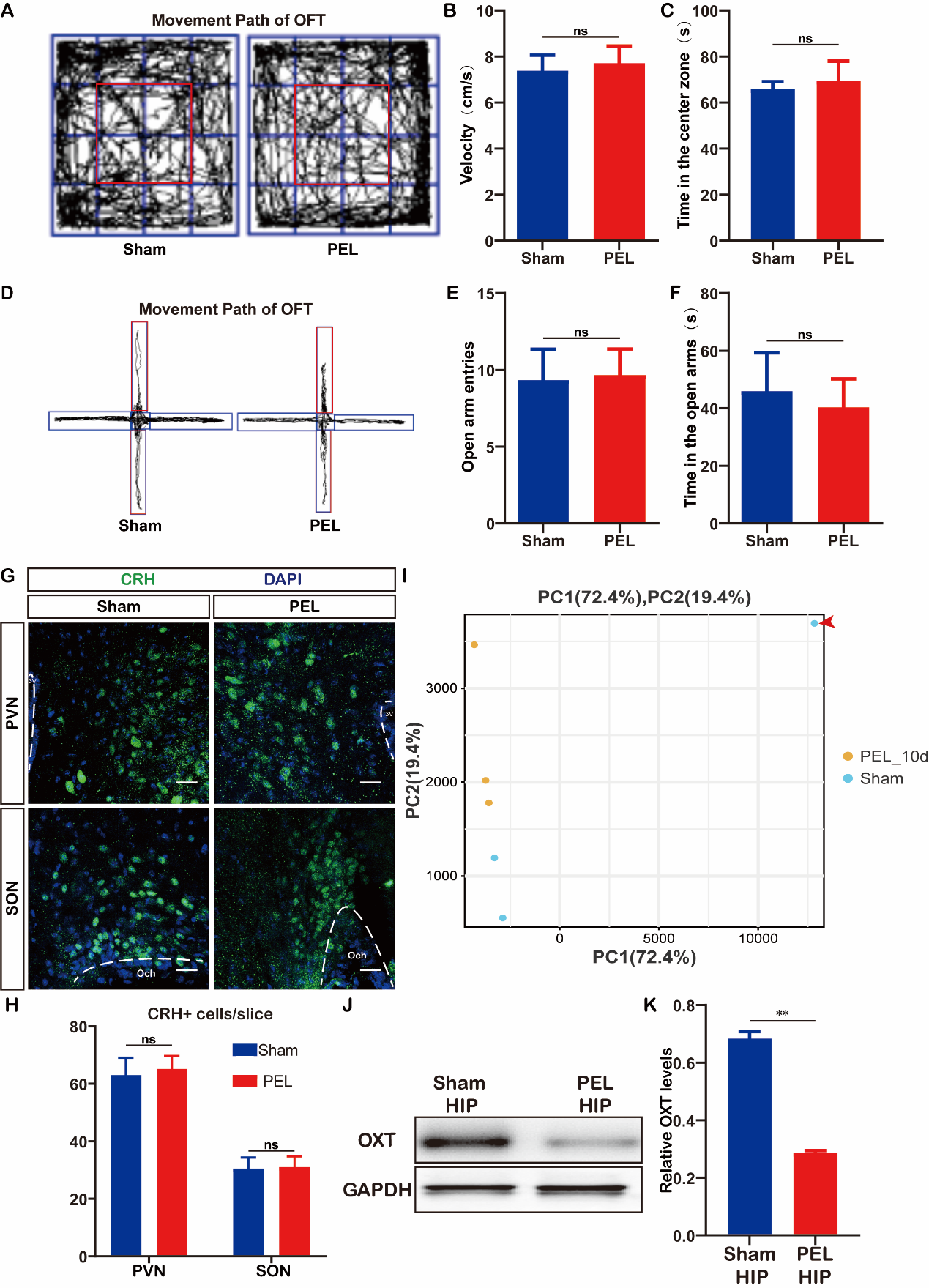


Figure S2 PEL treatment does not affect locomotion and anxiety levels in mice.

(A) Trajectories of PEL-treated group（n=6）and Sham group（n=6） in the OFT test. The area in red is the center area.

(B) Quantitative analysis of velocity in the OFT test. (t=0.3221, df=10 *p*= 0.754.)

(C) Quantitative analysis of time in the center zone in the OFT test. (t=0.3923, df=10 *p*= 0.7031.)

(D) Trajectories of PEL-treated group（n=6）and Sham group（n=6） in the EPM test. The area in red is the open arm

(E) Quantitative analysis of open arm entries in the EPM test. (t=0.1270, df=10, *p*=0.9015.)

(F) Quantitative analysis of time in the open arms in the EPM test. (t=0.3368, df=10, *p*= 0.7432.)

(G) CRH neuronal was not lost after PEL.

(H) Quantification results of CRH neurons (n=6, PVN: t=0.6959, df=10, *p*= 0.5024; SON: t=0.2255, df=10 *p*= 0.8261)

(I) PCA maps of hypothalamus sequencing in Sham and PEL mice. Red arrow is outlier.

(J,K) Immunoblotting confirmed the decrease of OXT in hippocampal tissues of mice after PEL surgery (n=6,t=15.30, df=10, *p*<0.01)

Data were analyzed by unpaired t test (B, C, E, F, H, K). Data are expressed as mean± SEM. Compared to PEL-treated group, ***P* <0.01, ****P* <0.001. OFT: open field test; EPM: elevated plus maze


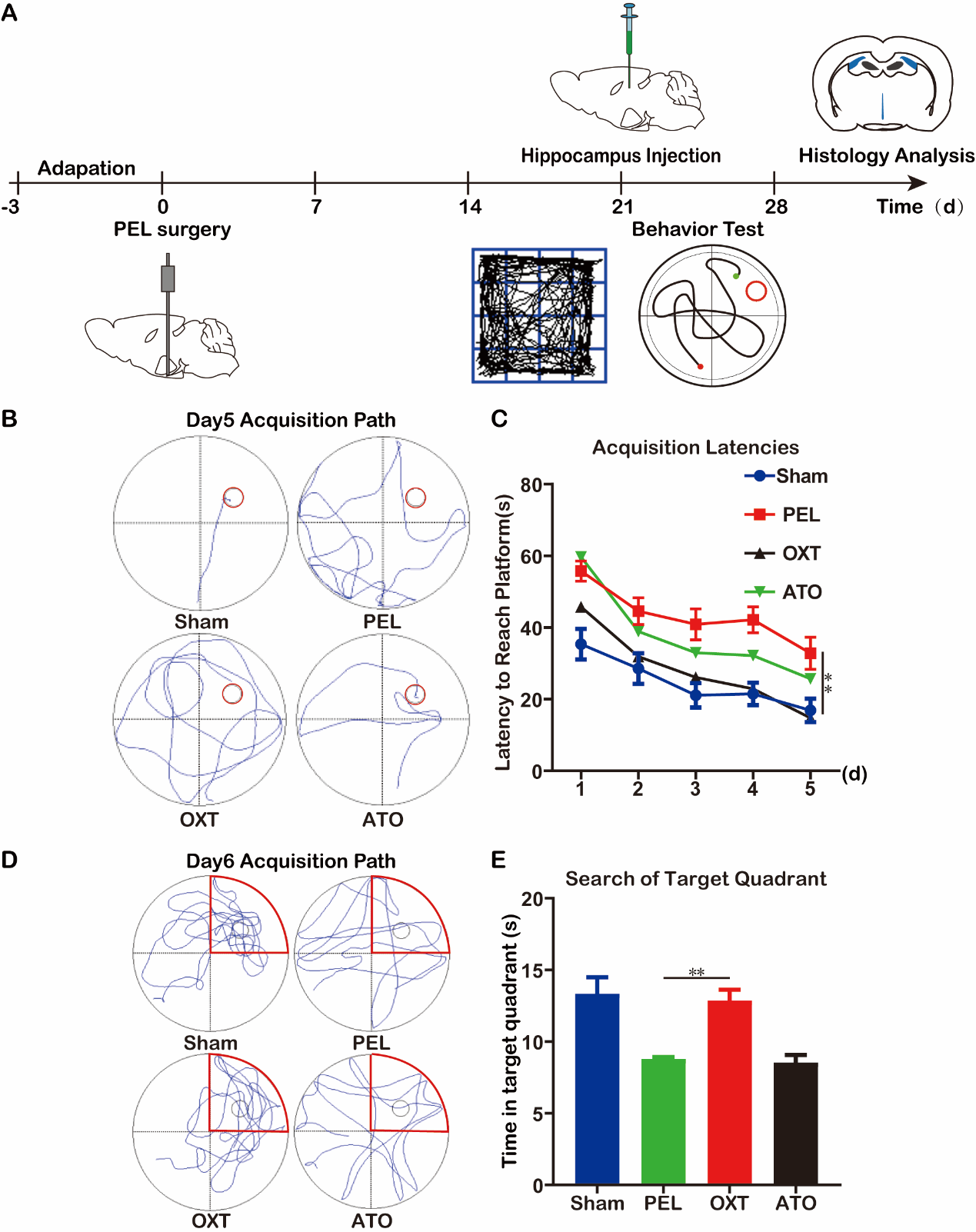


Figure S3 Intrahippocampal injection of OXT significantly improves PEL-induced cognitive dysfunction

(A) The entire experimental protocol of this study.

(B) Trajectories of Sham group（n=6）,PEL-treated group（n=6）, OXT-supplemented group（n=6）, and ATO group（n=6） in the MWM place navigation experiment

(C) Quantitative analysis of latency in the MWM place navigation experiment. (F (3.784, 348.1) = 32.74 *p*<0.01.)

(D) Trajectories of Sham group（n=6）,PEL-treated group（n=6）, OXT-supplemented group（n=6）, and ATO group（n=6） in the MWM spatial exploration experiment. The area in red is the area around the platform, and the activity time of mice in the shaded part is calculated.

(E) Quantitative analysis of swimming time around the platform in the MWM spatial exploration experiment. (F (3, 20) = 11.9, *p*<0.01.)

Data were analyzed by two-way ANOVA (B) and 1-way ANOVA followed by LSD multiple comparisons test (D). Data are expressed as mean± SEM. Compared to PEL-treated group, ***P* <0.01,. MWM: Morris Water Maze; OXT: oxytocin; ATO: Atosiban.


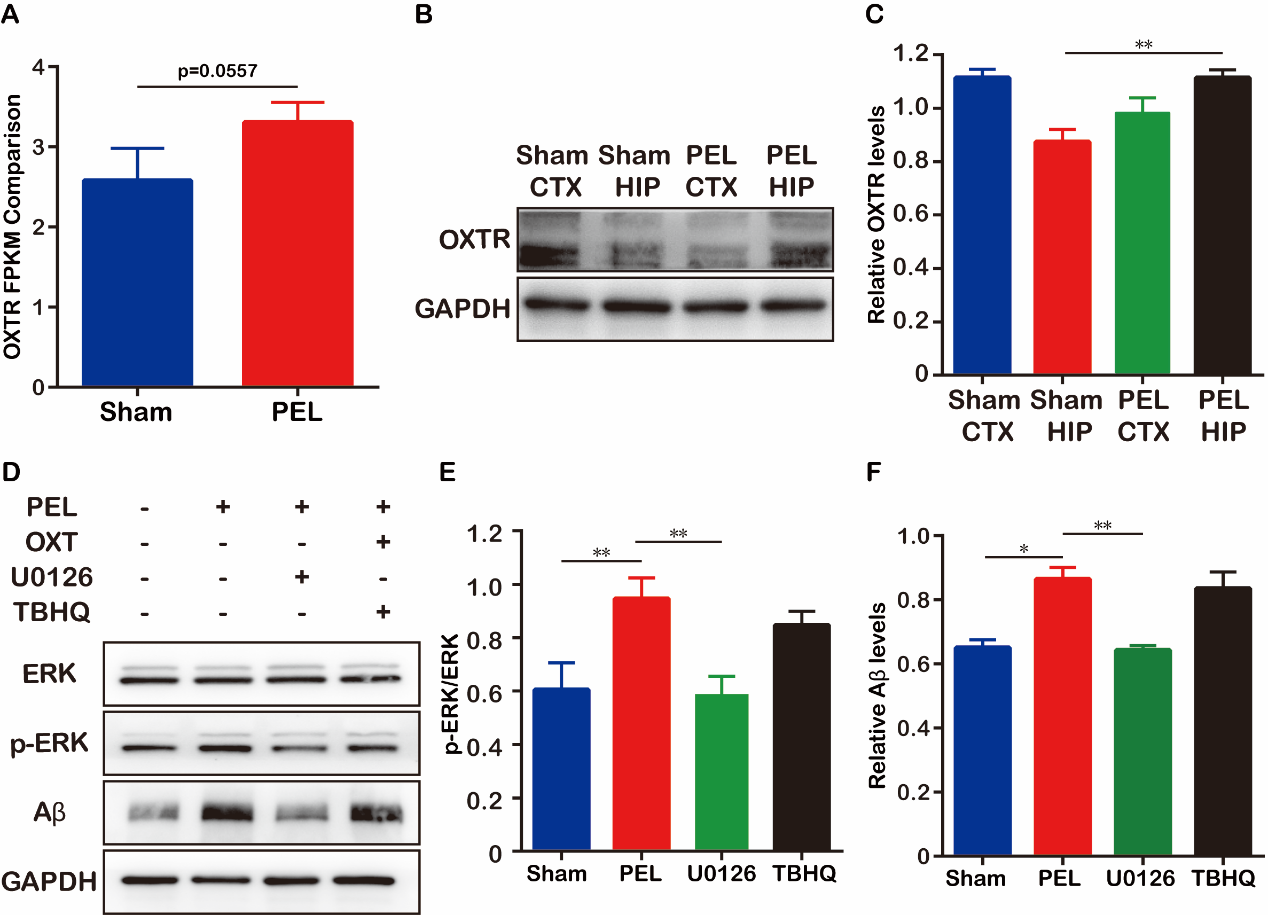


Figure S4 Validation of hippocampal OXTR signaling and ERK signaling pathway

Quantitative results of elevated OXTR FPKM values in the hippocampal region after PEL surgery (A n=3 each group, t=2.671, df=4, *p*=0.0557). Immunoblotting confirmed the elevated expression of OXTR in hippocampal tissue of mice after PEL surgery (B, C n=6,Kruskal-Wallis statistic=17.15, *p*<0.01.).Immunoblotting confirmed that U0126 attenuates ERK hyperphosphorylation in hippocampal tissue of mice after PEL surgery (D, E n=6 Kruskal-Wallis statistic= 17.86, *p*<0.01.).Immunoblotting confirmed that U0126 attenuated Aβ expression in hippocampal tissue of mice after PEL surgery (D, F n=6,Kruskal-Wallis statistic= 17.55, *p*<0.01.).

Data were analyzed by unpaired t test（A）and Kruskal-Wallis test (C, E, F). Data are expressed as mean± SEM. Compared to Sham, ***P* <0.01.
